# Supplementary figures and images for: Assessing the low complexity of protein sequences via the low complexity triangle
Source: PLoS One. 2020 Dec 30;15(12):e0239154. doi: 10.1371/journal.pone.0239154 (PMC7773278; doi:10.1371/journal.pone.0239154)

a) Globular (PDB)

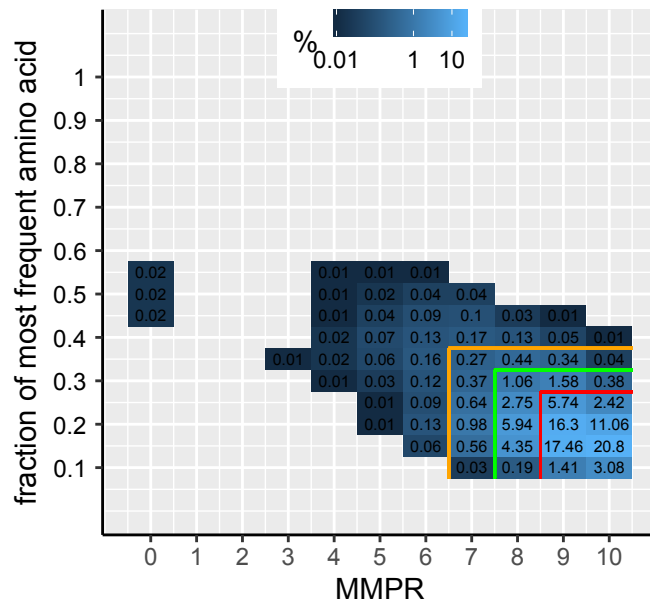

b) Low Complexity

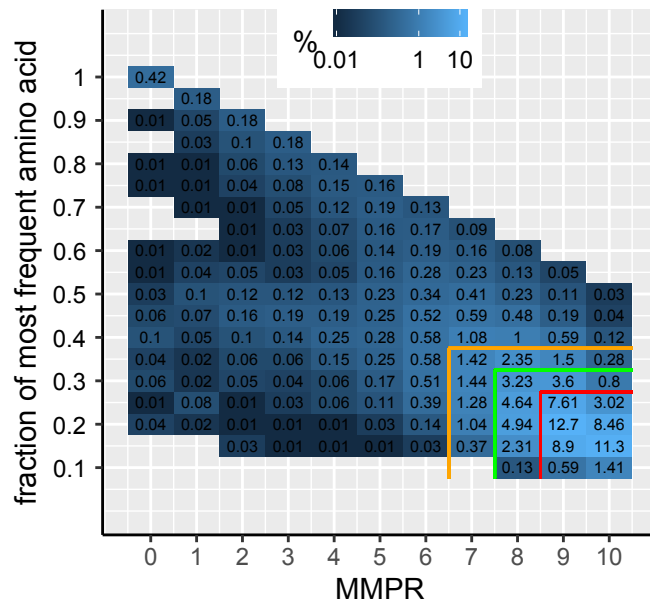

c) Disorder (DisProt)

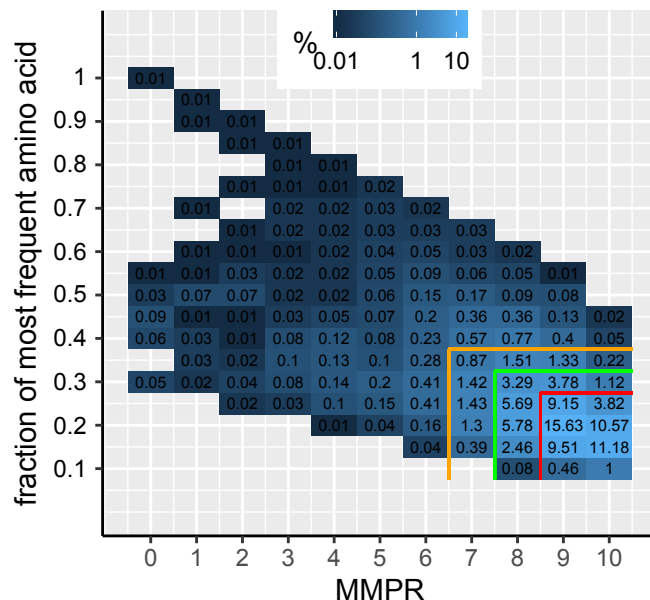

d)

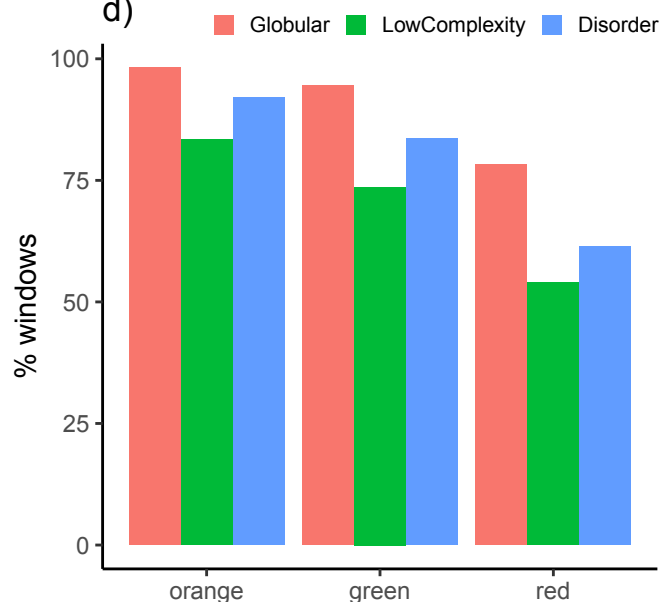

Supplement: S1 Fig — a) 1000 randomly selected globular proteins, from the PDB database, b) 100 manually-curated low complexity proteins, c) disordered regions from DisProt release 2020_06, d) percentage of windows covered in the red, green and orange regions per dataset. MMPR = Minimum number of Mutations to Perfect Repeats. Window abundance shown in log10 scale. Window length = 20 amino acids. (PDF) [file pone.0239154.s001.pdf]

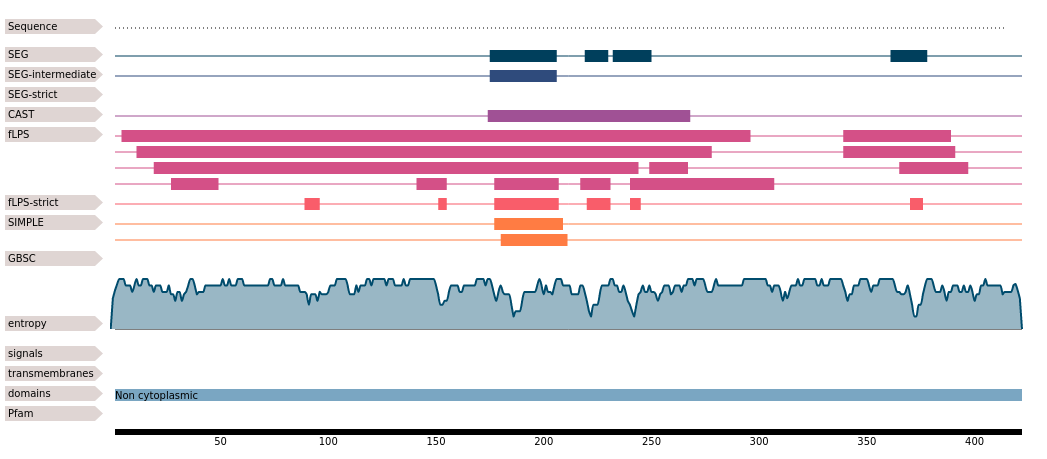

Supplement: S2 Fig — (PNG) [file pone.0239154.s002.png]

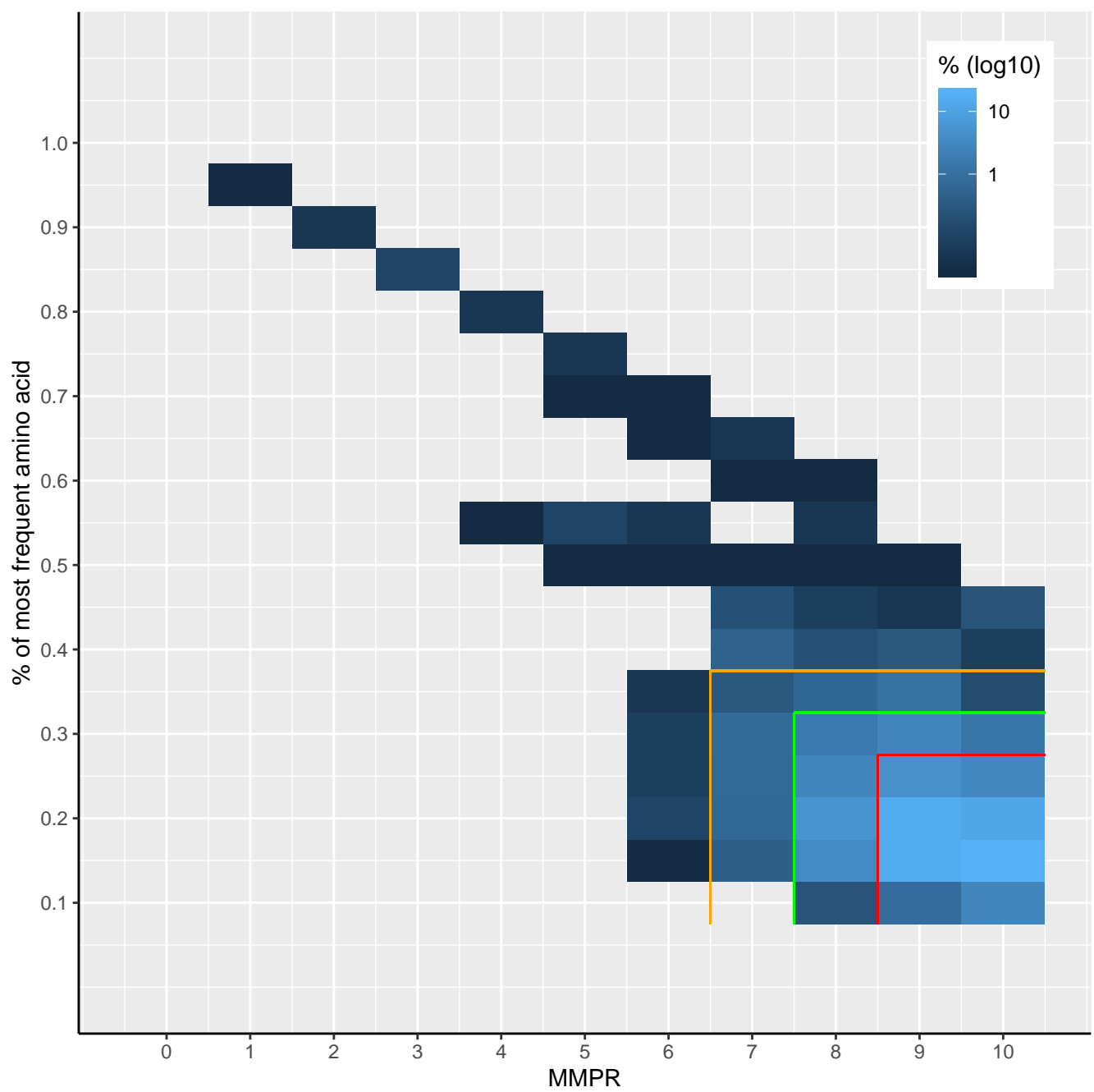

Supplement: S8 File — (ZIP) [file pone.0239154.s010.zip › lct/test.fasta.pdf]
